# Future-ready antimicrobials: harnessing organic acids to override Ca2+-driven barrier failure during Salmonella infection

**DOI:** 10.1099/mic.0.001735

**Published:** 2026-07-15

**Authors:** Igori Balta, Ioan Pet, Nicolae Corcionivoschi, Lavinia Stef

**Affiliations:** 1 Faculty of Bioengineering of Animal Resources, University of Life Sciences King Mihai I from Timisoara, 300645 Timisoara, Romania; 2 Bacteriology Branch, Veterinary Sciences Division, Agri-Food and Biosciences Institute, Belfast BT4 3SD, Northern Ireland, UK; 3 Academy of Romanian Scientists, Ilfov Street, No. 3, 050044 Bucharest, Romania

**Keywords:** E-cadherin, intracellular Ca^2+^, occludin, organic acids, *Salmonella *Typhimurium, ZO-1

## Abstract

Organic acid mixtures are widely employed as feed additives to suppress enteric pathogens, yet their mechanisms of host protection at the intestinal epithelium remain unclear. *Salmonella* Typhimurium is a major cause of foodborne illness and mainly affects the caecal epithelium in poultry, where disruption of epithelial junctions enables bacterial adhesion, invasion and systemic spread. In this study, we examined the effects of a sub-inhibitory concentration of a commercial organic acid mixture, AuraShield (As) 0.25%, on *S*. Typhimurium SE10/72 infection in primary chicken caecal epithelial cells (PECC). Infected PECC showed decreased E-cadherin levels, altered zonula occludens-1 (ZO-1) and occludin mRNA expression and higher intracellular Ca²^+^, indicating junctional disruption and increased vulnerability to invasion. Treatment with 0.25% As significantly reduced bacterial adhesion and invasion, restored E-cadherin mRNA and protein expression along with its membrane localization and shifted ZO-1 and occludin transcript levels towards those of uninfected controls. Simultaneously, intracellular Ca²^+^ levels decreased towards baseline. These findings indicate that As protects the intestinal epithelium by maintaining E-cadherin–mediated adherens junctions, supporting tight junction gene expression and modulating Ca²^+^-dependent signalling during *Salmonella* infection. This research advocates the use of organic acid mixtures as host-directed interventions to strengthen the gut barrier and restrict pathogen colonization in poultry.

## Introduction

The current wave of ‘organic acid blend’ interventions is moving beyond a single-variable narrative of lowering pH to decline *Salmonella* levels toward a multi-axis biology in which sublethal exposures reshape bacterial virulence programmes, biofilm phenotypes and host epithelial responses. Recent investigations have illustrated this transition, as evidenced by a poultry-relevant *in vitro* biopsy model, suggesting that an organic acid blend can reduce *Salmonella* attachment and preclude invasion while concurrently dampening epithelial inflammation and restoring barrier function, thereby implicating intracellular calcium regulation as a potential host-side lever [1]. Furthermore, a recent functional analysis of >20 short-chain carboxylic acids showed that antimicrobial activity depends on intrinsic molecular properties and environmental pH, clarifying why ‘same acid class’ does not mean ‘same biological outcome’ [2].

One of the earlier conceptually valuable directions is the demonstration that certain organic-acid mixtures can meaningfully alter host epithelial integrity and inflammatory signalling, not just bacterial counts [3]. Cell and tissue models offer unusually clear mechanistic insight because they can co-measure pathogen invasion, epithelial injury (LDH release, TEER) and cytokine pathways under controlled dosing. As exemplified by a previous mechanistic study, organic acid mixtures (maltodextrin, citric acid, malic acid, citrus extract and olive extract) restored tight junction (TJ) integrity in *Salmonella enterica*-infected Madin–Darby Canine Kidney epithelial cells, as measured by increased TEER and higher expression of zonula occludens-1 (ZO-1) and occludin [3]. In chicken enteroids, wild-type *S*. Typhimurium disrupted ZO-1 distribution, whereas a non-invasive mutant did not, linking barrier injury to invasive virulence rather than to LPS exposure alone [4]. Similarly, in bovine ileal organoid-derived monolayers, invasive *Salmonella* Dublin caused redistribution of E-cadherin and a marked decline in TEER, indicating that junctional disorganization is a conserved epithelial consequence of invasive salmonellae [5]. A recent study introduces a particularly provocative host-side observation suggesting that an organic-acid blend can reduce intracellular Ca^2+^ levels in epithelial cells and biopsies and restore TEER, suggesting a calcium-linked barrier mechanism (not yet mechanistically resolved) [1].

We have previously shown that organic acid mixtures do far more than curb *Salmonella* virulence; they reprogram the host–pathogen interaction itself, simultaneously disarming the bacterium and fortifying the epithelial barrier. Remarkably, the AuraShield (As) formulation (5% maltodextrin, 1% sodium chloride, 42% citric acid, 18% sodium citrate, 10% silica, 12% malic acid, 9% citrus extract and 3% olive extract) not only reduces intracellular Ca²^+^ and oxidative stress in infected primary chicken caecal epithelial cells (PECC), but actively preserves tight-junction integrity and suppresses pro-inflammatory signalling, revealing a powerful dual-action mechanism with transformative potential for controlling enteric infections [1]. Beyond this, we now hypothesize that the As formulation exerts a host-directed protective effect by lowering infection-induced intracellular Ca²^+^ flux, thereby preventing the loss of E-cadherin and stabilizing tight-junction gene expression (ZO-1 and occludin). Consequently, this Ca²^+^-dependent mechanism is expected to reduce *Salmonella* adhesion and invasion in PECC cells significantly.

## Methods

### Bacteria, primary caecal epithelial cells and antimicrobials

*S. enterica* serovar Typhimurium SE10/72 from laboratory collection and the PECC (laboratory stocks) were cultured as previously described using the previously established sub-inhibitory concentration of natural antimicrobials [1]. After 2 days (at the second passage), wells with 70–80% surface coverage were used in subsequent experimental procedures. By including the previously established sub-inhibitory concentration of 0.25% As in this assay, we have also assessed if this working concentration affects the integrity of the epithelial cells. Briefly, PECC cells grown in the presence of 0.25% As or in the absence of As were grown for 36 h and snap-frozen in liquid nitrogen until use. RNA was isolated using the RNeasy Plus Mini Kit (Qiagen, United Kingdom). Cells were sampled at 12 h, 24 h and 36 h. All assays were performed in parallel. The RNA was reverse transcribed using the Transcriptor First Strand cDNA Synthesis Kit (Roche) according to the manufacturer’s protocol. The mRNA levels were determined by quantitative reverse transcription PCR (RT-PCR) using the QuantNova SYBR Green PCR Kit (Qiagen, United Kingdom) on a LightCycler 96 (Roche). The E-cadherin primers are included in Table 1. The housekeeping gene, GAPDH, was used as a control. The antimicrobial mixture, As, contained the following ingredients: 5% maltodextrin, 1% sodium chloride, 42% citric acid, 18% sodium citrate, 10% silica, 12% malic acid, 9% citrus extract and 3% olive extract (wt/wt). Environtech Dublin, Ireland, supplied the antimicrobial mixture.

**Table 1.** Primers used in this study

Gene name	Primer sequence (5′−3′)
*E-cadherin*	F: GACAGGGACATGAGGCAGAA
	R: GCCGTGACAATGCCATTCTC
*GAPDH*	F: TGCTGCCCAGAACATCATCC
	R: ACGGCAGGTCAGGTCAACAA
*ZO-1*	F: TCTGCACAGTGAGGTTGGCT
	R: GGCTGTCCTGCATCGGTGT
*Occludin-1*	F: TGCTTTTGCCCAAGCAGGAA
	R: TGTGGGAGAGGCACCAGTTG
*RPLP0*	F: TCACGGTAAAGAGGGGAGGTG
	R: CTTGCTCAGTCCCCAGCCTT

### Infection assay and immunohistochemistry

PECC were cultured in DMEM supplemented with 10% FBS and grown to 80–90% confluence in 24-well plates for 22–24 h before infection. *S*. Typhimurium SE10/72 cultures were harvested, washed and adjusted to an OD_₆₀₀_ of ~0.3 (1×10^5^ c.f.u./ml) before addition to PECC at a m.o.i. of 100. Before infection, cells were washed and overlaid with medium containing 0.25% As where required. After infection, monolayers were washed and treated with 0.1% Triton X-100 to quantify cell-associated bacteria; serial dilutions were plated on TSAYE agar and incubated to enumerate colonies. Gentamicin (400 µg ml^−1^) was then added for 3 h to eliminate extracellular bacteria, after which cells were lysed and internalized bacteria quantified using the same plating method. All assays were performed in triplicate on three independent days [1]. For immunocytochemistry, *S*. Typhimurium SE10/72 was stained with 5-TAMRA-SE (Cat#, C2211, Thermo Fisher Scientific, UK), and infected PECCs were fixed, permeabilized and stained for E-cadherin using rabbit anti-E-cadherin (Cat# RAB02404, 1 : 100 dilution; Thermo Fisher Scientific, UK) followed by Alexa Fluor-488 secondary antibody (Cat# A32731TR, 1 : 1,000 dilution; Thermo Fisher Scientific, UK); nuclei were counterstained with DAPI (Cat# R37606, Thermo Fisher Scientific, UK), and images were captured using a Zeiss LSM 700 confocal microscope. Infection studies and the intracellular Ca²^+^ measurements were performed as previously described [1].

### Immunoblotting

The infected cells, treated or untreated with As, were boiled in SDS-PAGE loading buffer. Protein samples (40 µg) were run on SDS-PAGE and subsequently transferred to nitrocellulose membranes. The membranes were blocked with 5% dried milk in Tris-buffered saline and Tween-20 (20 mM Tris-HCl, 150 mM NaCl, 0.05% Tween-20) for 6 h at room temperature. After washing with 25 mM Tris buffer, the membranes were incubated with the respective primary antibody (Cat# RAB02404, 1 : 100 dilution; Thermo Fisher Scientific, UK) overnight at 4 °C. After thorough washing, the membranes were incubated with an HRP-conjugated secondary antibody solution for 1 h at room temperature. The membranes were washed with 25 mM Tris buffer (3X), and the blots were detected using an enhanced chemiluminescence reagent and exposed to photographic film (Kodak, Thermo Fisher, UK). Images were collected with the E-Gel Imager from Life Technologies.

### Statistical methods

Statistical analyses were performed using GraphPad software, version 11. In some cases, data were represented as mean±sd. *P*-values<0.05 were considered statistically significant. One-way ANOVA, Two-way ANOVA and Dunnett’s multiple comparisons tests were used depending on the power needed. Data distribution was assessed using the Shapiro–Wilk test to verify normality. Homogeneity of variances was evaluated using Levene’s test. Biological replicates are defined as independent bacterial cultures or independent cell culture experiments performed on different days. Technical replicates are defined as multiple measurements (e.g. replicate wells or readings) from the same biological sample.

## Results

### Organic acids mediate *S*. Typhimurium infection via host cell E-cadherin expression in PECC cells

**Fig. 1.**
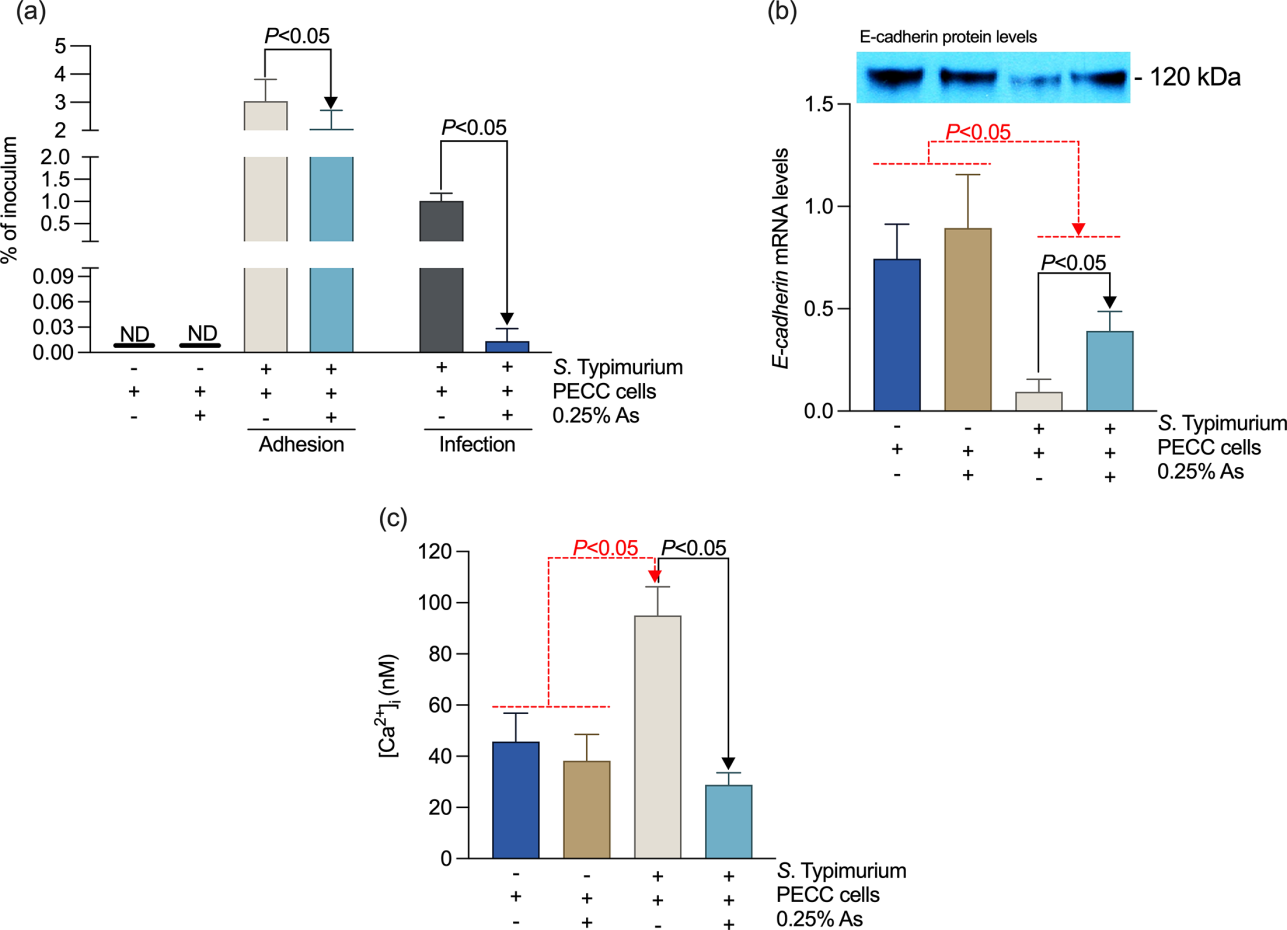
Organic acids in mixture (As) impair *S*. Typhimurium SE10/72 infection in PECC cells via E-cadherin expression and Ca^2+^ regulation. As reduces *S*. Typhimurium adhesion and invasion of PECC and restores E-cadherin expression via modulation of intracellular Ca²^+^. (**a**) Adhesion and invasion of *S*. Typhimurium in PECC cells in the absence or presence of 0.25% As. (**b**) Relative E-cadherin mRNA and protein expression levels in infected PECC cells with and without 0.25% As treatment. (**c**) Intracellular Ca²^+^ levels in PECC cells under four conditions: uninfected, uninfected+0.25% As, infected and infected +0.25% As. Data are presented as mean±sem from three independent experiments performed in triplicate. Statistical analysis was performed using Dunnett’s test for multiple comparisons; *P*<0.05 was considered significant and is indicated on the graphs.

*In vitro*, *S*. Typhimurium adheres (~3% of the inoculum) and infects (~1% of the inoculum) PECC cells (Fig. 1a). The organic acid mixture (0.25% As) interferes with both adhesion and infection by significantly reducing the number of bacteria that attach to or enter epithelial cells (*P*<0.05). The data presented indicate that the As mixture modulates the infection dynamics of *S*. Typhimurium in PECC cells by affecting host cell E-cadherin expression (Fig. 1b) at both the mRNA and protein levels through regulation of intracellular calcium (Fig. 1c) (*P*<0.05). Overall, these data suggest that treatment with 0.25% As significantly reduced bacterial adhesion and invasion and restored E-cadherin mRNA and protein levels. Additionally, a decrease in intracellular Ca^2+^ was noted under these conditions, emphasizing the connection between calcium signalling and epithelial barrier function during infection.

### Immunohistochemistry analysis

**Fig. 2.**
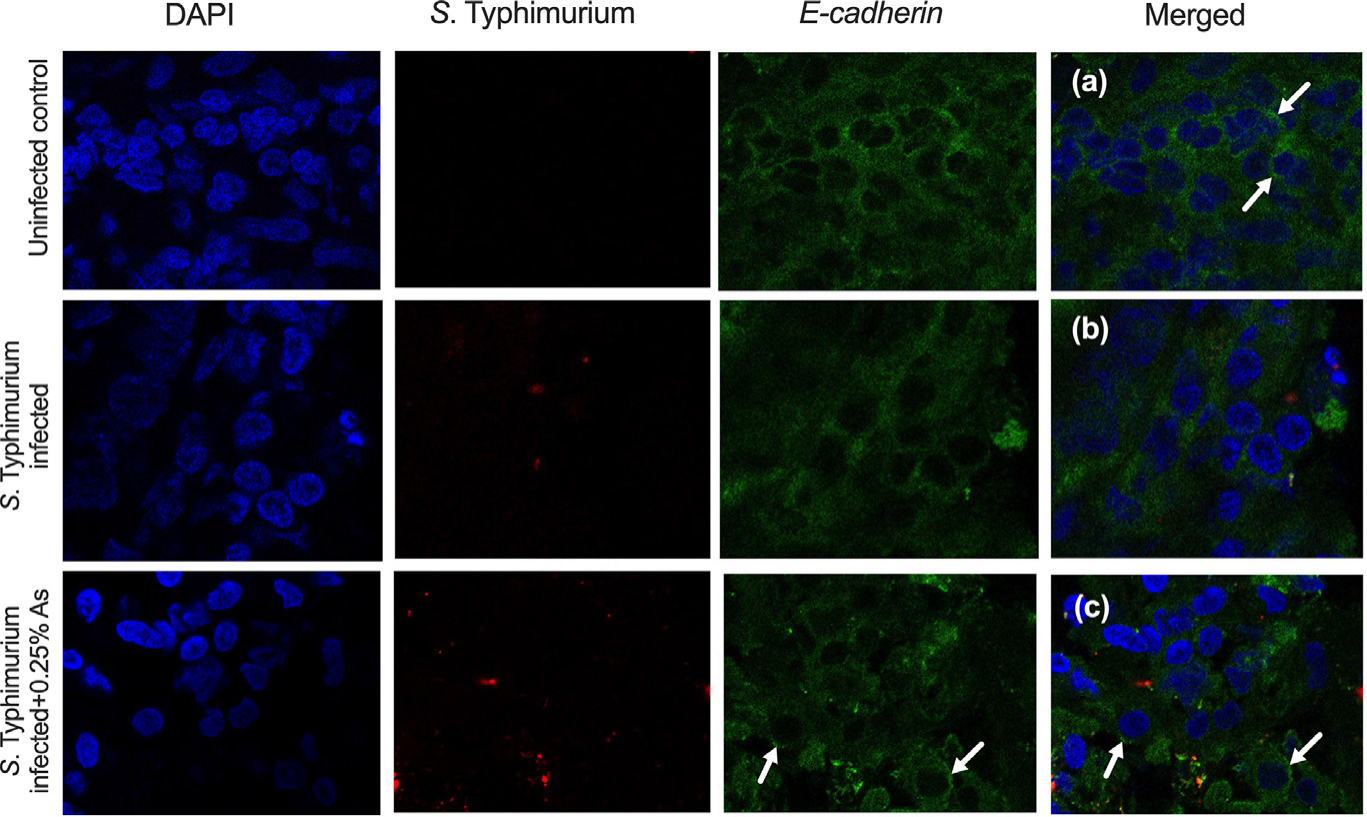
Immunofluorescent analysis of E-cadherin expression in PECC following *S*. Typhimurium SE10/72 infection and treatment with 0.25% As. (**a**) Uninfected PECC cells showing strong E-cadherin localization at the cell membrane surrounding the nuclei, indicating intact epithelial junctions. (**b**) PECC cells infected with *S*. Typhimurium exhibited reduced E-cadherin signal and disrupted membrane localization, consistent with compromised epithelial integrity. (**c**) Infected PECC cells treated with 0.25% As demonstrated restored E-cadherin membrane localization and improved junctional continuity. Nuclei are stained with DAPI (blue), E-cadherin with Alexa Fluor 488 (green) and bacteria with TAMRA (red). Images were acquired using a Zeiss LSM 700 confocal laser scanning microscope.

The impact of As treatment on E-cadherin expression in *S*. Typhimurium-infected PECC cells was investigated (Fig. 2). Immunohistochemistry (ICC) analysis showed the presence of the E-cadherin protein around the nuclei of uninfected PECC cells, as indicated by white arrows (Fig. 2a). The ICC staining for E-cadherin is significantly decreased in infected PECC cells (Fig. 2b), and treatment with 0.25% As restored E-cadherin localization at cell membranes, indicating improved epithelial integrity (Fig. 2c). We now cautiously suggest a possible link between the application of organic acids and the preservation of epithelial integrity during bacterial infection. Collectively, this evidence supports a protective mechanism whereby organic acids counteract pathogen-induced disruption in PECC cells.

### ZO-1 and occludin mRNA levels recover in the presence of 0.25% As

**Fig. 3.**
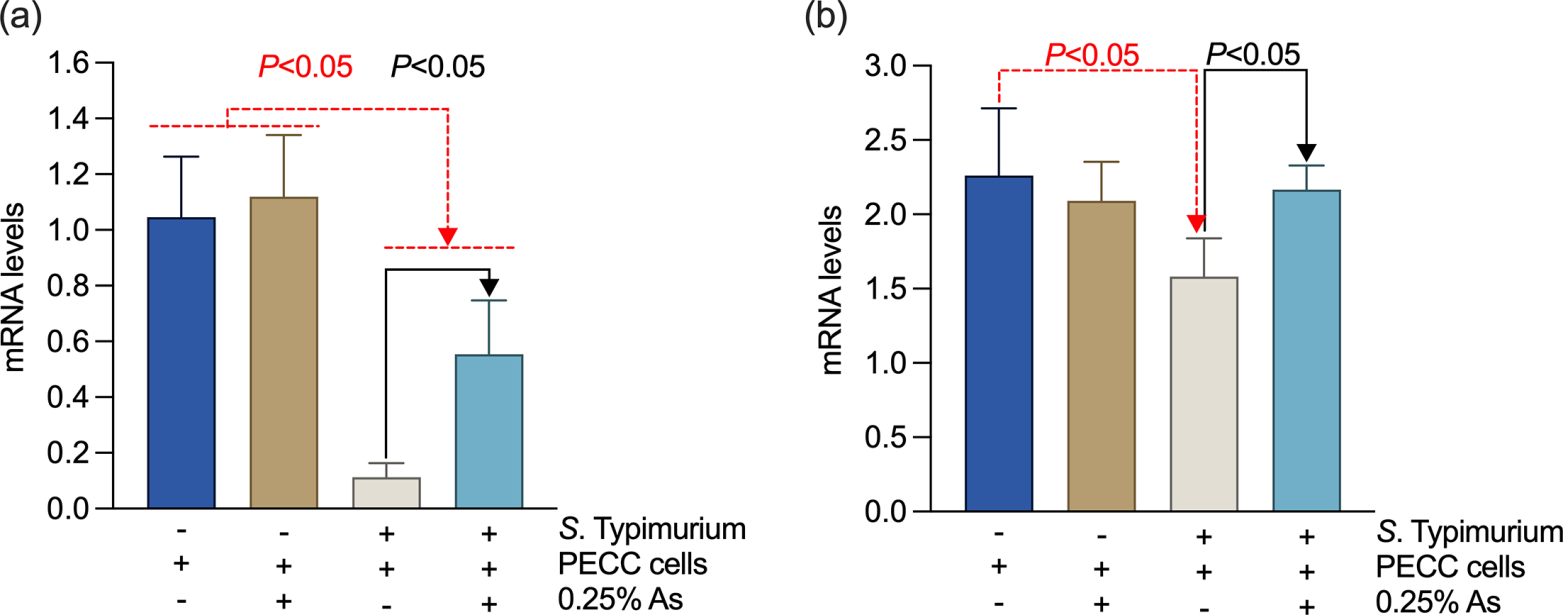
Effects of As on ZO-1 (**a**) and occludin (**b**) mRNA expression during *S*. Typhimurium infection of PECC. PECC cells were left uninfected, infected with *S*. Typhimurium SE10/72, or infected in the presence of 0.25% As. Relative mRNA expression levels of the TJ–associated genes ZO-1 and occludin were quantified by qRT-PCR using GAPDH and RPLP0 as housekeeping controls. Data are presented as mean±sem from three independent experiments performed in triplicate. Statistical analysis was carried out using Dunnett’s test for multiple comparisons; *P*<0.05 was considered statistically significant and is indicated on the graphs.

To further explore the effect of As on TJ structural proteins during *S*. Typhimurium infection of PECC cells, we measured ZO-1 and occludin mRNA levels. In uninfected cells, ZO-1 (Fig. 3a) and occludin (Fig. 3b) mRNA levels indicate the baseline TJ status of a healthy epithelium. Upon infection with *S*. Typhimurium, the expression of these genes changes, consistent with disruption of TJ architecture and increased epithelial permeability that favour bacterial adhesion and invasion. This infection-induced dysregulation demonstrates the pathogen’s ability to impair barrier function by affecting junctional components. When infected cells are treated with 0.25% As, ZO-1 and occludin mRNA levels shift towards those observed in uninfected controls (*P*<0.05). This partial or complete restoration of TJ gene expression indicates that the organic acid mixture helps preserve or re-establish epithelial barrier integrity under infectious stress. Together with the effects on E-cadherin and intracellular Ca²^+^ described in Figs1  4, these data support a model in which As protects the caecal epithelium by stabilizing multiple junctional components at the transcriptional level, thereby limiting *Salmonella*-mediated barrier disruption and subsequent invasion.

**Fig. 4.**
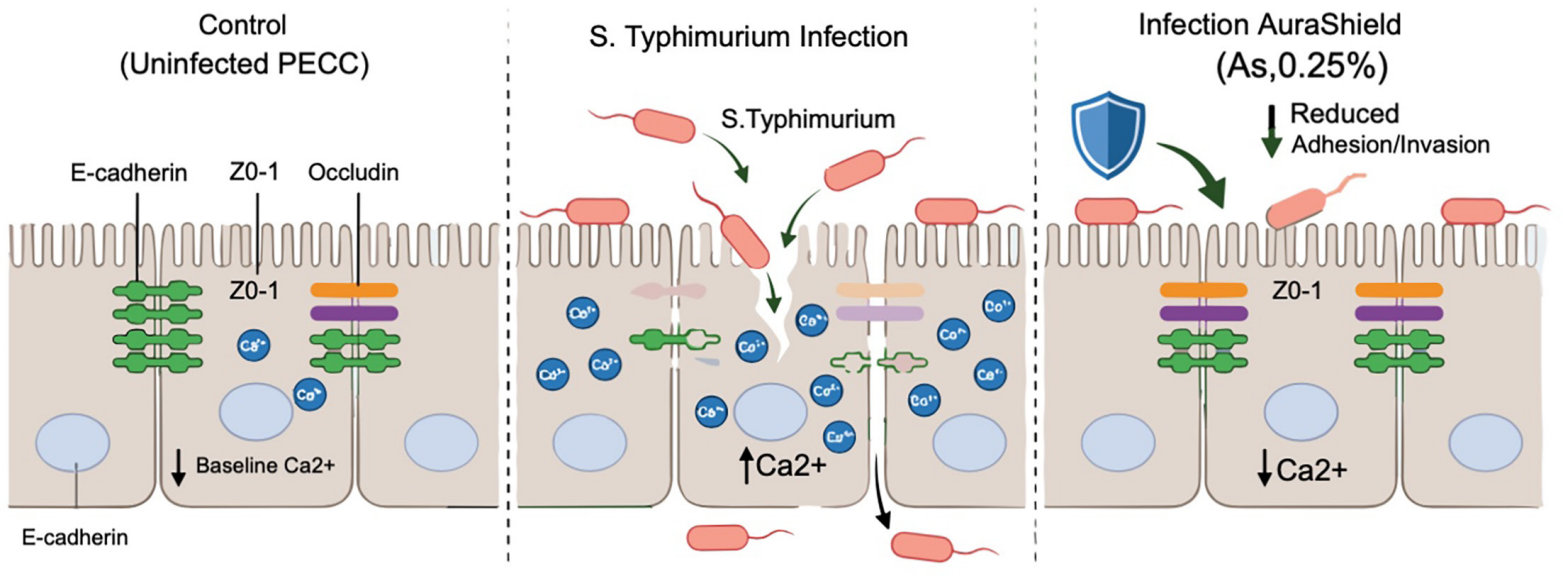
Proposed mechanism by which As protects PECC cells against *S*. Typhimurium infection. *S*. Typhimurium SE10/72 infection of PECC cells increases intracellular Ca²^+^ levels, leading to reduced E-cadherin mRNA and protein expression, loss of E-cadherin from the cell membrane and disruption of epithelial junctional integrity, which favours bacterial adhesion and invasion. In the presence of 0.25% As, intracellular Ca²^+^ levels decrease towards those of uninfected cells, E-cadherin transcription and protein expression are restored and E-cadherin localization at the cell membrane is maintained. This preservation of E-cadherin–mediated junctions reinforces the epithelial barrier. It significantly reduces *S*. Typhimurium adhesion and invasion in PECC cells, highlighting the host-directed protective role of organic acid mixtures.

## Discussion

Prior evidence indicates that cellular TJs have traditionally been regarded as independent complexes, even though their formation relies on cadherin- and nectin-based adhesions [6]. In the initial stages of junction formation, both non-epithelial and epithelial cells involve ZO-1 in the lateral aggregation of cadherin-catenin complexes and the clustering of occludin [7]. Crediting mixtures of organic acids with the ability to directly modulate TJ integrity is challenging and, at times, speculative; however, emerging evidence suggests that they can help regulate intestinal permeability, a key factor in protection against gastrointestinal diseases, nutrient imbalances and inflammatory conditions [8]. For example, citric acid, a component of As, promotes the expression of TJ proteins, including E-cadherin, thereby enhancing cell survival and immune function in fish [9]. In viral infections, it has been suggested that citric acid promotes the growth of beneficial bacteria (e.g. *Bifidobacterium* and *Lactobacillus*), strengthens the intestinal TJ barrier and bolsters intestinal immune function [10]. Additionally, this organic acid can mitigate feed ingredient-induced enteropathy in the distal intestine of juvenile turbot [11]. This involvement in disease pathogenesis is reflected in an imbalance in cell differentiation, and direct and indirect effects on the intestinal epithelial barrier have also been described for maltodextrin, another component of the As mixture [12].

More recently, we have shown that *S*. Typhimurium infection of PECC cells (chicken caecal primary epithelial cells) leads to increased intracellular Ca^2+^ and higher infection rates [1]. This short investigation suggests that the increase could be associated with reduced E-cadherin mRNA expression and that 0.25% As during infection could lower intracellular Ca^2+^, restore E-cadherin expression and significantly impair *S*. Typhimurium’s ability to adhere to and infect PECC cells. E-cadherin is a calcium-dependent transmembrane protein essential for cell adhesion and tissue integrity, primarily expressed in epithelial cells [13]. Bacterial infections can reduce E-cadherin expression and facilitate bacterial invasion by activating Ca^2+^-dependent proteases that cleave E-cadherin [14], thereby directly affecting cell-cell adhesion and signal transduction [15]. We have also repeatedly shown that mixtures of natural antimicrobials (organic acids) play a crucial role in preserving the integrity of the gut [16] and respiratory [17] epithelium by strengthening cellular TJs and reducing oxidative stress.

## Conclusions

This study describes the potential host-protective role of an organic acid mixture, demonstrating that at a sub-inhibitory concentration, As could reshape the outcome of *S*. Typhimurium SE10/72 infection (Fig. 4). By restoring E-cadherin, ZO-1 and occludin expression and by reducing infection-induced intracellular Ca²^+^, As prevents the cascade of junctional collapse that normally enables bacterial adhesion and invasion. This Ca²^+^-centred mechanism, paired with reduced oxidative stress, positions As not only as an antimicrobial additive but as a next-generation, host-directed defender of epithelial integrity. Together, these findings indicate that organic acid mixtures like As could serve as a tool to suppress pathogens. Organic acids have the potential to reengineer the host–pathogen interface itself, offering a powerful, antibiotic-free strategy for future control of enteric infections. However, we also need to acknowledge that assessing such mechanisms solely through molecular and protein biomarkers could limit the interpretation of our results.

## Abbreviations

As: AuraShield
ICC: immunohistochemistry
PECC: primary chicken caecal epithelial cells
RT-PCR: reverse transcription polymerase chain reaction
TJ: tight junction
ZO-1: zonula occludens-1
